# RV dyssynchrony in PAH as evidenced by slice to slice minimum volume timing

**DOI:** 10.1186/1532-429X-17-S1-P170

**Published:** 2015-02-03

**Authors:** Qiao Han, Stephan Philip Leonhardt Altmayer, Amit R Patel, Karima Addetia, Yuchi Han

**Affiliations:** Cardiology, University of Pennsylvania, Philadelphia, PA USA; Radiology, The University of Chicago Medicine, Chicago, IL USA

## Background

Pulmonary hypertension has been associated with right ventricular (RV) dyssynchrony. In patients with left side heart failure, treating left ventricular dyssynchrony has been shown to improve patient outcome. Previous studies have demonstrated that RV dyssynchrony was associated with RV dilation, interventricular septum diastolic flatterning and eccentric hypertrophy. We propose simple parameters from cine MRI that can potentially help quantify the complex movements without using special sequences or algorithms.

## Methods

### Study population

Four healthy subjects (2 females and 2 males) and 11 pulmonary arterial hypertension (PAH) patients (10 females and 1 male) were included in the study. Two patients had QRS duration of >120 ms.

### CMR imaging

The study population underwent cardiac MRI exams on 1.5 T units (Avanto Siemens, Erlangen, Germany or Phillips Achieva, Best, Netherlands). Cine short axis data were obtained using steady state free procession imaging with voxel size 1.67x1.67mm, repetition time 29.4ms, echo time 1.19ms for the Siemens unit, and voxel size 1.42x1.42mm, repetition time 2.49ms, echo time 1.25ms for the Phillips unit.

### CMR analysis

Short axis cine and RVOT images were imported into QMass (Medis, Laiden, the Netherlands). Endocardial contours were manually traced in all phases for the RV in QMass. Trabeculation was included in the contours, and RV structural information was subsequently calculated.

### Dyssynchrony

For each ventricle, we identified the global min volume phases, the min volume phase for each slice, and converted phase numbers into exact timing. We denoted the two timings as t_0,_ t_i_ respectively. We calculated two parameters to describe the levels of dyssynchrony present in different subjects: T_DEVslices_MinV_, the deviation of {t_i_} from t_0_, and T_SDslices_, the standard deviation of {t_i_}.

## Results

**Figure 1:**
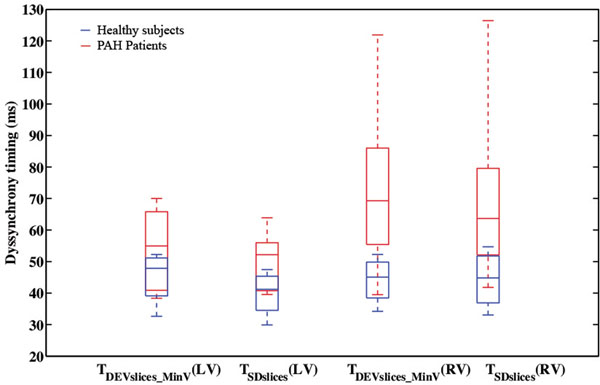
LV and RV Dyssynchrony evidenced by slice to minimal volume timing.

**Figure 2:**
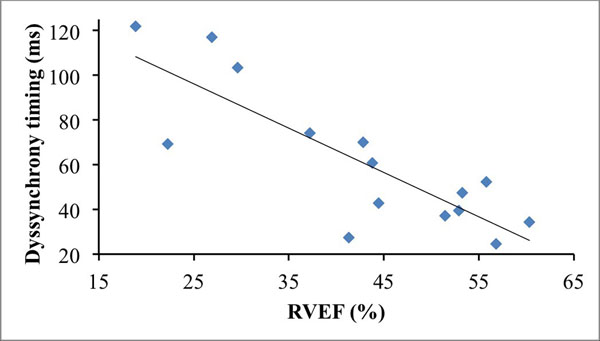
T_DEVslices_MinV_(RV) showed moderate correlation with RVEF (y = -1.97x + 145.41, R2 = 0.61).

The average LV ejection fraction (EF) for healthy subjects was 67.0±4.0%, and 60.3±6.5% for PAH patients. The average RVEF for healthy subjects was 54.5±6.7%, and 38.5±12.7% for PAH patients. RV septal wall bounce was observed in majority of our patient population. In the quantitative assessments for LV, RV, and two ventricles combined, PAH patients showed higher variation in the timing of contraction among individual slice positions as well as higher deviation from the minimal RV volume phase (T_DEVslices_MinV_) (Figure 1). Normal range of T_DEVslices_MinV_ for the RV is 34-52 ms and a subpopulation of the PAH population falls into this range. Other PAH patients have "dyssynchrony", including the two patients with prolonged QRS. The RV dyssynchrony parameters showed moderate correlations with RVEF (Figure 2).

## Conclusions

RV function is an important clinical measurement especially in the prognosis of pulmonary hypertension. We proposed simple parameters T_SDslices_ and T_DEVslices_MinV_ derived from cine CMR to describe RV dyssynchrony in PAH patients.

## Funding

CMREF: cardiovascular medical research and education fund.

